# Biochemical and hematological changes among anemic and non-anemic pregnant women attending antenatal clinic at the Bolgatanga regional hospital, Ghana

**DOI:** 10.1186/s12878-018-0121-4

**Published:** 2018-09-17

**Authors:** Benjamin Ahenkorah, Kwabena Nsiah, Peter Baffoe, Enoch Odame Anto

**Affiliations:** 1 Biochemistry and Hematology Units, Bolgatanga Regional Hospital, P.O. Box 26, Bolgatanga-Upper East Region, Ghana; 2 Department of Biochemistry and Biotechnology, Kwame Nkrumah University of Science and Technology, Kumasi, Ghana; 3 Obstetrics and Gynecology Unit, Bolgatanga Regional Hospital, P.O. Box 26, Bolgatanga-Upper East Region, Ghana; 4 Department of Biochemistry and Molecular Medicine, School of Medical and Health Science, University for Development Studies, Tamale, Ghana; 5 Department of Molecular Medicine, School of Medical Science, Kwame Nkrumah University of Science and Technology, Kumasi, Ghana; 6 Department of Medical Laboratory Technology, Royal Ann College of Health, Atwima-Manhyia, Kumasi, Ghana; 7 School of Medical and Health Science, Edith Cowan University, Perth, WA Australia

**Keywords:** Anemia, Pregnant women, Hematological, Biochemical, Iron deficiency

## Abstract

**Background:**

Anemia in pregnancy may not only be associated with maternal morbidity and mortality but can also be detrimental to the fetus. A definitive diagnosis of anemia is a pre-requisite to unravelling possible cause(s), to allow appropriate treatment intervention. It is hypothesised that measured hemoglobin (HGB), complemented by biochemical and other hematological parameters would enhance anemia diagnosis.

**Methods:**

This was a cross-sectional study among 400 pregnant women comprising 253 anemic and 147 non-anemic pregnant women, attending an antenatal clinic at Bolgatanga Regional Hospital, Ghana. Venous blood was collected and hemoglobin genotype, complete blood count and biochemical parameters [ferritin, iron, total iron binding capacity (TIBC), transferrin saturation (TfS), C-reactive protein (CRP) and bilirubin] were determined. Thick blood films were prepared for malaria parasitemia, while early morning stool and midstream urine samples were examined for enteric and urogenital parasites, respectively.

**Results:**

There were significantly reduced levels of HGB (*p* < 0.0001), HCT (*p* < 0.0001), MCV (*p* < 0.0001), iron (0.0273), ferritin (*p* = 0.018) and transferrin saturation (0.0391) and increased WBC (*p* = 0.006), RDW (*p* = 0.0480), TIBC (*p* = 0.0438) and positivity of CRP in anemic, compared to non-anemic pregnant women. Anemic women were associated with increased proportion of hemoglobinopathies (AS, SS and SC), *Plasmodium falciparum, Schistosoma hematobium* and intestinal parasite infections.

**Conclusion:**

Anemic pregnant women are associated with a significant derangement in hematological and iron indices that implicate iron deficiency. This was influenced by hemoglobinopathies and parasitic infections.

## Background

Anemia is the most prevalent nutritional deficiency problem during pregnancy. Iron deficiency anemia is the leading cause of anemia in most developing countries [1]. Anemia and iron deficiency anemia are often used interchangeably and the prevalence of anemia taken to be the same as that of iron deficiency [1]. It has been reported that 56% of pregnant women in low income countries are affected [2], in contrast to 18% in high income countries [3].

In Ghana, anemia has been attributed to poor bioavailability of iron in the diet, which is due to the low intake of foods that enhance absorption of iron [4–6]. During pregnancy, there is disproportionate increase in the plasma volume, relative erythrocyte number, leading to a fall in hemoglobin concentration [7]. Moreover, the iron requirement generally exceeds the amount provided in the diet [8]. Intestinal iron absorption increases during pregnancy but becomes poor in cases of associated parasitic infectious diseases, such as hookworm and roundworm infestations [9].

Anemia during pregnancy is associated with a number of maternal and fetal disorders including the risks of preterm births, low birth weight babies, perinatal mortality and intrauterine growth retardation [10, 11]. Formation of fetal hemoglobin and myoglobin requires iron. Fetal iron is obtained from maternal stores, which progressively leads to depletion of iron in the mother. Therefore, adequate iron is required for a successful pregnancy and fetal outcomes [12].

A number of hematological [13, 14] and iron-related indices [15] have been used in the diagnosis of anemia in pregnancy. Although serum ferritin concentration of less than 12 μg/L is usually used as an indicator of iron deficiency [16], in tropical countries, a cut-off of 30 μg/L has been recommended as being the best indicator of deficient iron stores [17]. Elevated ferritin level, serum transferrin, transferrin receptor (TfR), TIBC, erythrocyte sedimentation rate, and C-reactive protein concentrations, and reduced serum iron concentrations and transferrin saturation are usually associated with anemia of chronic disease [18].

The routine assessment of anemia has been based on hemoglobin levels of < 11 g/dl. However, other red cell indices such as MCV, MCH, MCHC and RDW have been widely employed in anemia diagnosis [18]. A reduced Hb, MCV below the lower reference limit for normal and an increased RDW above the upper reference limit for normal have been associated with microcytic anemia in pregnancy [19].

Although the anemic condition is progressive throughout pregnancy and is known to alter the red cell indices and iron status, few studies have studied both factors in pregnancy. Some studies, apart from measuring these factors independently, did not simultaneously consider the effect of Plasmodium parasite, intestinal parasites and *Schistosoma hematobium* infestation. It is against this background that we determined the changes in iron status and red cell indices, along with the presence of sickle cell and parasitic infections, among anemic and non-anemic pregnant women, visiting an antenatal clinic at the Bolgatanga Regional Hospital, Ghana.

## Methods

### Study design and setting

This hospital-based cross-sectional case-control study was conducted in the Obstetrics and Gynecology Department of the Regional Hospital Bolgatanga (RHB), Ghana, West Africa from May, 2013 to May, 2014.

### Study population and participant recruitment

A total of 400 pregnant women, comprising 253 anemic pregnant women with hemoglobin concentration < 11 g/dl using WHO criteria [20], were considered as cases and 147 non-anemic pregnant women of hemoglobin concentration > 11 g/dl, were purposively recruited as control.

### Inclusion and exclusion criteria

The study included all pregnant women attending their first antenatal care, of ages ranging from 15 to 48 years. Pregnant women in need of emergency care or having an at-risk pregnancy such as gestational diabetes, pre-eclampsia and eclampsia, were excluded. Antenatal pregnant women reporting for repeat visits during the study period and subjects who had been confirmed to be HIV positive were also excluded from the study.

### Specimen collection and processing

Five (5) milliliters (mls) of participants’ venous blood samples were drawn for hematological and biochemical analysis, between the hours of 8:00 am and 9:00 am. About 2mls were collected into BD vacutainers, containing EDTA for determination of hematological parameters and 3mls into BD Vacutainers with SST II Advance semi-separator gel, for determination of biochemical parameters. Samples in SST were centrifuged at 3000 rpm for 10 min and serum samples were aliquoted into cryotubes and stored at -80^o^ C until assay. Also, about 2 drops (6 μl) of blood were collected on a slide for the preparation of thick blood film to detect the presence of malaria parasites, according to the protocol described by Ahenkorah et al. [21].

About two grams (2 g) of early morning stool and 10 mls midstream early morning urine samples were also collected into sterile containers. The urine was used for the determination of *Schistosoma hematobium* ova/cyst and the stool for the determination of intestinal parasites. The collected samples were transferred in a cold box to the Biochemistry and Hematology Laboratory of RHB, for the required investigations.

### Hematological assay

Full blood count was performed using the Sysmex KX-21 N Automated Hematology Analyzer (Sysmex Corporation Kobe, Japan) Whole Blood Mode. The parameters for the full blood count determination were; WBC, HGB, HCT, RDW, MCHC and MCV. Routine quality control checks were run on control specimen within specified limits per the manufacturer’s instructions. Hematology analyzer was calibrated per the instructions of the manufacturer when there was a change of reagent. The presence of Hb variants was detected on a hemolysate prepared from EDTA sample on cellulose acetate paper at pH 8.5, using an electrophoresis set-up (Beijing Liuyi Instrument Factory, China).

### Biochemical assay

Serum ferritin was measured, using the AXSYM, MEIA quantitative technique. Serum iron and UIBC were assayed by the modified method of Henry (1984), using the BT3000 Plus Chemistry Analyser (Biotecnica Instruments, Rome, Italy). Transferrin saturation index was calculated by dividing serum iron by TIBC and expressing the result as a percentage. Bilirubin determination was based on the modification of Tietz’s method (1994). Daily quality control checks were run on control specimen within specified limits per the manufacturer’s instructions. Clinical chemistry analyzer was calibrated per the instructions of the manufacturer when there was a change of reagent.

The CRP Latex test was used to determine the level of inflammation. It is a rapid slide agglutination test for the qualitative and semi-quantitative detection of C-reactive protein in serum. The reagent, containing particles coated with specific anti-human C-reactive protein antibodies, agglutinates in the presence of CRP in the patient’s serum.

### Malaria parasite screening

Parasitemia was determined using both the parasite density and plus (+) system. All the thick blood smears were stained with 10% Giemsa and examined under the × 100 oil immersion objective lens of a light microscope. For parasite density determination, the number of asexual parasites was counted against 200 leucocytes, where an average leucocytes count of 8000/μL was assumed. The blood smear was considered negative when 200 high power fields had been examined without visible parasite [22].

For the plus (+) system counting of parasite, the results were categorised as follows:

1–9 parasites per 100 microscopic fields (+); 10–99 parasites per 100 microscopic fields (++); 1–9 parasites per microscopic field (+++); more than 10 parasites per microscopic field (++++). The examination of the blood film for malaria parasites was done by two certified microscopists independently who were blinded to each other’s results [23].

### Stool and urine analysis

The formol-ether concentration method was used in the preparation of stool samples for microscopy and detection of intestinal parasite.

The urine sedimentation technique was used to detect the presence of *S. hematobium* ova. About 10 mls of urine was filtered using paper filters and the egg/ova count was recorded per 10 mls of urine.

### Statistical analysis

Data were entered into Microsoft Excel worksheet. Results were presented as mean ± standard deviation (SD) and frequency (percentage) and geometric mean (95% CI), where necessary. The Fischer’s exact test or Chi-square (X^2^) was used to assess the statistical significance of categorical variables. Unpaired sample t-test was used to compare between two means of continuous variables for normally distributed data and Mann-Whitney U test was used to compare between two medians of continuous variable for non-parametric variables. *P*-value less than 0.05 was considered statistically significant. Analysis was performed using GraphPad Prism 5 Project software (GraphPad software, San Diego California USA, www.graphpad.com).

## Results

Table 1 shows the biochemical profiles of study participants. There was no statistically significant difference between the mean ages of anemic pregnant women (27.53 ± 5.31 years), compared to non-anemic pregnant women (28.02 ± 4.97 years) (*p* = 0.309). Anemic pregnant women had a significantly lower mean HGB, HCT, MCV, MCHC than their non-anemic counterparts (*p* < 0.0001). Conversely, there was a significantly higher mean WBC and RDW amongst anemic pregnant women (*p* < 0.05). There were statistically significant increased levels of TIBC in anemic pregnant women, compared to the non-anemic pregnant women. The anemic pregnant women also had significantly lower median levels of serum ferritin (*p* = 0.0180), iron (*p* = 0.0273) and %TfR saturation (*p* = 0.0391). Bilirubin (total, direct and indirect) levels were lower in the anemic than the non-anemic pregnant women (*p* > 0.05). A higher proportion of anemic pregnant women had MCV < 80 fl (16.9% vs 5.4%; *p* = 0.0115), RDW > 15.0% (15.0% vs. 3.4%; *p* = 0.0052), serum iron < 40 μg/dl (18.6% vs. 6.1%; *p* = 0.0092), ferritin < 12 ng/ml (16.2% vs. 6.1%; *p* = 0.0400) and TIBC> 500 μg/dl (15.4% vs. 3.4%; *p* = 0.0015), compared to non-anemic pregnant women.

**Table 1:** Hematological and biochemical profile stratified according to anemic and non-anemic pregnant women

Parameters	Anemic	Non-anemic	*p*-value
	(*n* = 253)	(*n* = 147)	
Maternal age (years)	27.53 ± 5.31	28.02 ± 4.97	0.309
Hematological profile			
HGB (g/dl)	9.31 ± 1.34	11.89 ± 0.80	< 0.0001
WBC (/μL)	10.26 ± 1.68	6.13 ± 1.39	0.0006
HCT (%)	29.08 ± 3.34	35.43 ± 4.76	< 0.0001
MCHC (g/dl)	31.88 ± 2.02	33.43 ± 1.58	< 0.0001
MCV (fl)	78.72 ± 9.02	85.94 ± 8.32	< 0.0001
RDW (%)	16.87 ± 5.95	14.79 ± 3.55	0.048
Biochemical profile			
Serum ferritin (ng/ml)^a^	19.7 (15.2–94.0)	29.3 (21.4–106)	0.0180
Serum iron (μg/dl)^a^	88.3 (73.9–182)	152.7 (81.0–201)	0.0273
TIBC (μg/dl)^a^	369 (118.0–532.0)	302 (93–379)	0.0438
TfS (%)^a^	20.7 (6.6–26.4)	24.6 (8.1–27.5)	0.0391
Total Bilirubin (μmol/l)	18.09 ± 6.63	18.64 ± 6.33	0.564
Direct Bilirubin (μmol/l)	5.45 ± 2.13	5.82 ± 2.37	0.249
Indirect Bilirubin (μmol/l)	12.79 ± 6.08	13.26 ± 6.06	0.593
MCV (< 80 fL)^b^	43 (16.9%)	8 (5.4%)	0.0115
RDW (> 15%)^b^	38 (15.0%)	5 (3.4%)	0.0052
Serum iron(< 40 μg/dl)^b^	47 (18.6%)	9 (6.1%)	0.0092
Ferritin (< 12 ng/ml)^b^	41 (16.2%)	9 (6.1%)	0.0400
TIBC(> 500 μg/dl)	39 (15.4%)	5 (3.4%)	0.0015

*TIBC* Total iron binding capacity, *TfS* Transferrin saturation, *HGB* hemoglobin, *WBC* White blood cells, *HCT* Hematocrit, *MCHC* Mean corpuscular hemoglobin concentration, *MCV* Mean corpuscular volume, *RDW* Red cell distribution width. *p* < 0.05 was considered statistically significant different

Values are presented as mean ± standard deviation, ^a^median (interquartile range). ^b^frequency (percentages)

Table 2 shows the association between anemia and parasitemia. Higher proportion of women with anemia had malaria parasitemia, and intestinal parasitic infections. There was a statistically significant association between anemia and malarial parasitemia (*p* = 0.0111), as well as intestinal parasite (*p* = 0.0152). The proportion of + 1 (20.6% vs. 9.5%) and 2++ (4.0% vs. 0.7%) malaria parasite was significantly higher in anemic, compared to non-anemic. There was a significantly increased geometric mean of ring trophozoite of *Plasmodium falciparum* among anemic pregnant women (2159 pa/μl blood), compared to their non-anemic counterparts (809.4 pa/μl blood) (*p* = 0.0487). The proportion of intestinal parasite 1+ among anemic women (27.7%) was significantly higher, compared to non-anemic (18.4%) counterparts (*p* = 0.0152). All schistosomiasis infections were found in anemic participants, giving a proportion of 0.4% (1/253) each of S. *hematobium* (1+) and *S. mansoni* (1+) infection.

**Table 2:** Association between anemia and malaria infection, intestinal parasite and Schistosomiasis among pregnant women

	Anemic (*n* = 253)	Non-anemic (*n* = 147)	Statistics	*p*-value
Characteristics			X^2^ value, df	
Plasmodium falciparum			6.455, 1	0.0111
Not seen	186 (73.5%)	132 (89.8%)		
1+	52 (20.6%)	14 (9.5%)		
2++	10 (4.0%)	1 (0.7%)		
Parasite density(pa/ul)#	2159 (926.2–7205.3)	809.4 (479.3–941.5)		0.0487
Intestinal parasite			8.371, 2	0.0152
Not seen	183 (72.3%)	120 (81.6%)		
1+	70 (27.7%)	27 (18.4%)		
*Schistosomiasis*			–	–
Not seen	251 (99.2)	147 (100.0%)		
S. *haematobium* (++)	1 (0.4%)	–		
*S. mansoni* (++)	1 (0.4%)	–		

Values are presented as frequency (percentage); #: geometric mean (confidence interval). X^2^: Chi-square value; df: degree of freedom

Also, from Table 3, the proportions of AA and CC genotypes between the anemic and non-anemic pregnant women were not statistically significantly different (*p* > 0.05). A significantly higher percentage of the non-anemic (21.8%) had AC genotype, compared to the anemic (9.4%) (*p* = 0.0327). On the other hand, a significantly higher percentage of AS genotype was found among the anemic (16.2%), compared to non-anemic (4.1%) pregnant women (*p* = 0.0081). Additionally, SC 4.7% (12/253) and SS genotypes 0.4% (1/253) were found in only the anemic pregnant women.

**Table 3:** Hemoglobin genotypes of the pregnant women with and without anemia

Hb Genotype	Anemic *n* = 253	Non-Anemic *n* = 147	*p*-value
AA	166 (65.6%)	105 (71.4%)	0.5428
AC	24 (9.5%)	32 (21.8%)	0.0327
AS	41 (16.2%)	6 (4.1%)	0.0081
CC	9 (3.6%)	4 (2.7%)	0.4448
SC	12 (4.7%)	–	–
SS	1 (0.4%)	–	–

Values are presented as *n* (%). Comparisons between proportions of anemic and non-anemic groups were performed using Fischer’s exact test. *p* < 0.05 was considered statistically significant different

**Fig. 1:**
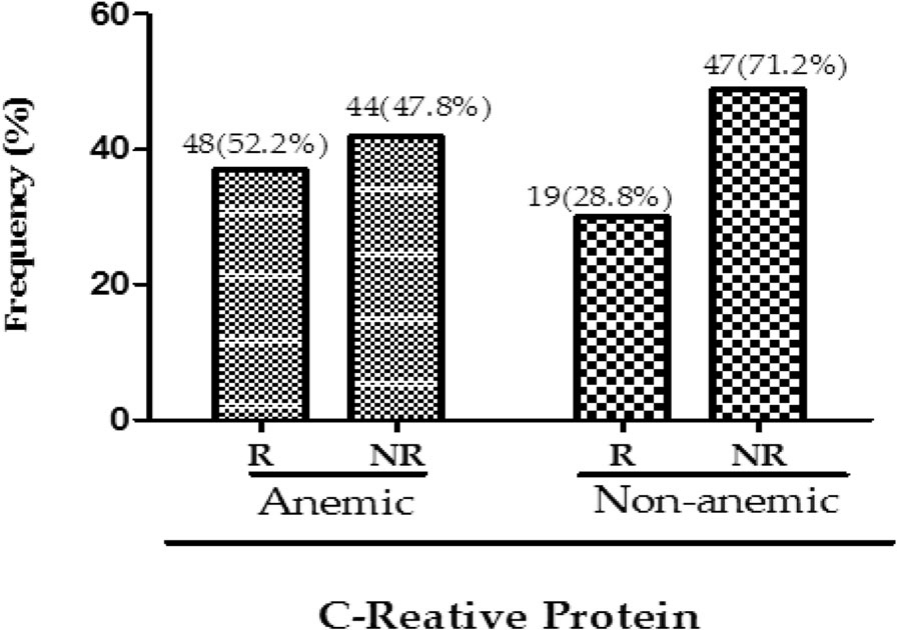
C - reactive protein of study participants. R: Reactive; NR: Non-reactive. Values in parentheses represent proportions of the responses between the two groups of women

Subjects who had anemia had a more positive response to C-reactive protein 52.2% (48/92) than the non-anemic pregnant women 28.8% (19/66) **(**Fig. 1).

## Discussion

This study determined the hematological and biochemical changes, along with the occurrence of sickle cell and parasitic infections among anemic and non-anemic pregnant women.

In this study, hematological indices such as Hb, HCT, MCHC and MCV levels were lower in anemic pregnant women, compared to their non-anemic counterparts. This is consistent with several studies [24–26] among anemic pregnant women. The mean Hb level of 9.31 g/dl observed among the anemic pregnant women fell within the moderate anemia (7.0 < Hb < 9.9 g/dl) category, as per WHO classification criteria for anemia [20]. The reduced HCT can be attributed to the reduced Hb concentration [27]. Additionally, a reduced HCT may arise from increase in plasma volume and hormonal changes during pregnancy, which cause hemodilution and fluid retention [28].

This study also observed a significantly low mean MCV level among anemic pregnant women, as 16.9% of them had MCV < 80 fL. A reduced MCV below the lower reference limit, suggests microcytic anemia or iron deficiency anemia [25]. Our finding that MCV levels were reduced in anemic pregnant women is consistent with that of Erhabor et al. who had a similar study [29] among anemic pregnant women in Sokoto, Nigeria. Erhabor and colleagues linked their findings to microcytic hypochromic anemia. The low MCV in our study is further supported by a significant rise in RDW level in the anemic pregnant women, which was above the cut-off point of 15%. The significantly higher proportion of anemic pregnant women who had RDW > 15% is consistent with the observation made by Tasneem et al. [13].

The significantly lower serum iron, ferritin and transferrin saturation among anemic pregnant women supports the finding by Nuzhat et al. [30]. Low levels of iron, ferritin and transferrin are suggestive of iron deficiency anemia. Our study observed that the proportion of anemic pregnant women with low iron (< 40μg/dl) and ferritin (< 12 ng/ml), were significantly higher among anemic pregnant women; this is another evidence of iron deficiency anemia [15]. We also observed a significantly higher TIBC in the anemic pregnant women. A higher than normal TIBC is an indication of iron-deficiency anemia. Our result corroborates that of Bleyere et al. [31], in a study among pregnant women in Cote d’lvoire. The probable explanation to the high TIBC levels among anemic pregnant women could be the reduced iron and % transferrin saturation [32].

The present study shows non-significantly lower levels of bilirubin among anemic pregnant women. The low bilirubin levels among the anemic pregnant women probably rules out hemolysis of the red cells.

Increased WBC could be as a result of increased inflammation and/or defensive immune response to infection [33]. This study observed a non-significantly increased levels of total WBC count among anemic pregnant women, compared to non-anemic women. Luppi [34] observed an increasing level of total lymphocyte count throughout pregnancy, which could be due to maternal body’s attempt to build up immunity. Our present study observed that majority of anemic pregnant women had parasitic infections like malaria and schistosomiasis; hence the elevated WBC is more likely to be attributed to these infections [33]. Although the mean WBC of anemic pregnant women in this study was elevated, it was within the reference range.

In the present study, the positive response of anemic pregnant women to C-reactive protein was 52.2% while non-anemic pregnant women showed 28.8%. A study by Mburu et al. [35] indicated that C-reactive protein levels > 6 ng/ml is indicative of increased inflammatory response, due to parasitic infections and or hemoglobinopathies.

From this study, Hb genotypes AS, SC and SS may have contributed to the higher number of anemic pregnant women due to the increased proportion of sickle cell trait and disease in the participants. Anemia is a major feature of sickle cell disease due to defective haemoglobin structure [36]. Anemia in pregnancy complicated by sickle cell disease or trait had been reported by Desai et al. [37]

In our previous study, an increased proportion of *Plasmodium falciparum* malaria and intestinal helminthes infections were observed among anemic pregnant women, compared to non-anemic pregnant women. Our current study supports our previous study [21] that anemic pregnant women reported with an increased proportion of *Plasmodium falciparum, Schistosoma hematobium* and intestinal parasites. This confirms the explanation that malaria and intestinal parasite infections coexist with micronutrient deficiencies, culminating in anemia [38].

The main strength of this study is the fact that despite working from a less resourced setting, we have been able to combine the measurement of biochemical and hematological parameters, unlike many other studies, where the two types of tests had been done independently. These measurements were done concurrently on same subjects, whereas in other studies, different subjects were used.

Despite these strengths, there were some limitations of our study. The use of single slide for parasite detection, cross-sectional nature of the study, inability to analyse all samples for CRP and the general lack of reference intervals specific to the local condition in Bolgatanga, which necessitated the comparison of the results of this study with data from countries whose socio-demographic variables vary from our local setting. Other micronutrients such as vitamin A, folate, cyanocobalamin, and zinc were not assessed in this study. Their influence on the burden of anemia in this setting can therefore become a subject for further scientific investigation.

## Conclusion

Anemic pregnant women are associated with some changes in hematological and iron indices including significantly reduced Hb, HCT, MCV, iron, ferritin and transferrin saturation and increased WBC, RDW, TIBC and positivity of CRP. These changes could have been influenced by a higher proportion of hemoglobinopathies, *Plasmodium falciparum, Schistosoma hematobium* and intestinal parasite.

## Abbreviations

CHRPE-KNUST/KATH: Committee on Human Research, Publication and Ethics of Kwame Nkrumah University of Science and Technology and Komfo Anokye Teaching Hospital
CRP: C-reactive protein
EDTA: Ethylenediaminetetraacetic acid
HCT: Hematocrit
HGB/Hb: Hemoglobin
IRB-NHRC: Institutional Review Board of the Navrongo Health Research Centre
MCH: Mean Corpuscular Hemoglobin
MCHC: Mean Corpuscular Hemoglobin Concentration
MCV: Mean Corpuscular Volume
MEIA: Microparticle Enzyme Immunoassay
PCV: Packed Cell Volume
RBC: Red Blood Cell
RDW: Red Cell Distribution Width
RHB-ANC: Regional Hospital Bolgatanga Antenatal Clinic
SST: Serum Separator Tube
TfS: Transferrin Saturation
TIBC: Total Iron Binding Capacity
UIBC: Unbound Iron Binding Capacity
WBC: White Blood Cell
